# Vitisin A Outperforms Cyanidin-3-O-Glucoside in Triglyceride Reduction by Modulating Hepatic Lipogenesis and Fatty Acid β-Oxidation

**DOI:** 10.3390/ijms26041521

**Published:** 2025-02-11

**Authors:** Yawen Li, Xusheng Li, Jia Liu, Pallavi Jayavanth, Weibin Bai, Rui Jiao

**Affiliations:** Department of Food Science and Engineering, College of Life Science and Technology, Jinan University, Guangzhou 510632, China; liyawen22@mails.ucas.ac.cn (Y.L.); lixusheng1016@163.com (X.L.); 13484879941@163.com (J.L.); pallavijayavanth@gmail.com (P.J.); baiweibin@163.com (W.B.)

**Keywords:** pyranoanthocyanin, vitisin A, triglyceride, fatty acid synthesis, β-oxidation

## Abstract

Pyranoanthocyanins exhibit greater bioactivity compared to monomeric anthocyanins, yet the lipid-lowering effects of pyranoanthocyanin Vitisin A, a primary derivative found in aged red wines, have not been extensively studied in vivo. This study evaluated the triglyceride-lowering effects of Vitisin A and its anthocyanin counterpart Cyanidin-3-O-glucoside (C3G) in both free fatty acid -induced HepG2 cells and high-fat diet-fed ApoE^-/-^ mice, with a focus on their roles in lipid metabolism. In vitro, Vitisin A significantly reduced triglyceride levels and lipid accumulation in HepG2 cells compared to C3G at equivalent concentrate. In vivo, dietary supplementation with 100 mg/kg of Vitisin A reduced body weight gain and plasma triglyceride levels by 19.6% and 29.5%, respectively, whereas no significant effects were observed with C3G. Mechanistically, Vitisin A markedly inhibited hepatic de novo lipogenesis (DNL) by activating the AMPK/ACC signaling pathway and downregulating FASN expression. Concurrently, Vitisin A enhanced fatty acid β-oxidation more robustly than C3G by upregulating CPT-1A via AMPK/SIRT1/PGC-1α and PPAR-α/PGC-1α pathways. Both Vitisin A and C3G driving peroxisomal β-oxidation of very-long-chain fatty acids. In summary, Vitisin A demonstrated superior triglyceride-lowering effects compared to C3G, primarily through dual mechanisms of inhibiting hepatic DNL and enhancing fatty acid β-oxidation.

## 1. Introduction

Hypertriglyceridemia, a prevalent lipid disorder, is strongly associated with early-onset coronary artery disease, cerebrovascular disease, peripheral arterial disease, non-alcoholic fatty liver disease (NAFLD), and diabetes [1,2]. Triglycerides (TGs) serve as the primary form of fatty acid transport and storage in cells and plasma, with the liver playing a central role in fatty acid metabolism [3,4,5]. Hepatic steatosis occurs when fatty acid input, including free fatty acid (FFA) uptake and de novo lipogenesis (DNL), exceeds output via β-oxidation and very-low-density lipoprotein (VLDL) secretion [6,7]. TG accumulation in the liver reflects the dynamic interplay of these processes, with DNL and fatty acid oxidation serving as critical regulatory mechanisms [8,9,10].

Red wine, a globally popular alcoholic beverage, owes its color to anthocyanins, a group of water-soluble pigments with potent antioxidant and anti-inflammatory properties [11]. Red wine contains over 2000 milligrams of anthocyanins per liter [12]. These bioactive compounds confer hepatoprotective effects by modulating lipid storage and fatty acid metabolism [13,14]. The excellent bioactivity of cyanidin-3-O-glucoside (C3G) has been widely documented in published research [15,16]. However, the application of C3G was limited due to the unstable structure during the food process and digestion. Generally, anthocyanins are sensitive to environmental factors, such as light, heat, pH, and oxygen. The degradation and color fading of anthocyanins affect the sensory quality of food and reduce its bioactivity [12].

Pyranoanthocyanins, formed during red wine fermentation and aging, are chemically stable anthocyanin derivatives characterized by enhanced color intensity and antioxidant capacity [17]. Vitisin A, the main anthocyanin derivative in aged wine, forms through the reaction between pyruvate and anthocyanins [18]. Our previous research demonstrated that vitisin A surpasses cyanidin-3-O-glucoside C3G in hypocholesterolemic activity in vitro; however, its effects on plasma lipid profiles in vivo remain unexplored [17]. This study aims to evaluate and compare the lipid-lowering effects of vitisin A and C3G in ApoE^−/−^ mice on a high-fat diet, focusing on their impact on lipid metabolism.

## 2. Results

### 2.1. Vitisin A Decreased Intracellular TG Levels and Lipid Accumulation in HepG2 Cells

**Figure 1:**
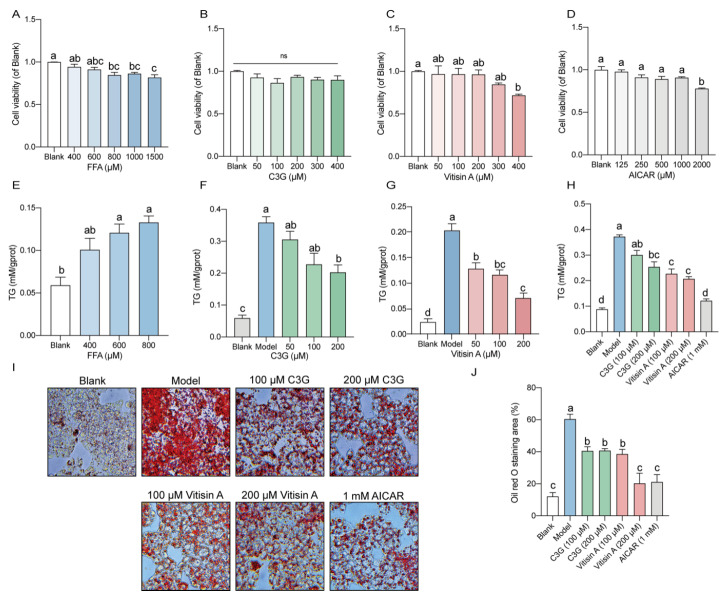
Effects of FFA, C3G, vitisin A, and AICAR with various concentrations on TG and Oil Red O staining in HepG2 cells. Cells were incubated with different concentrations of (**A**) FFA (0, 400, 600, 800, 1000, and 1500 μM), (**B**) C3G (0, 50, 100, 200, 300, and 400 μM), (**C**) vitisin A (0, 50, 100, 200, 300, and 400 μM), and (**D**) AICAR (0, 125, 250, 500, 1000, and 2000 μM) for 24 h. The cell viability of blanks was set as 1. Cells were cultured with (**E**) FFA with various concentrations for 24 h; (**F**) 600 μM FFA and different concentrations of C3G (50, 100, and 200 μM) for 24 h; (**G**) 600 μM FFA and different concentrations of vitisin A (50, 100, and 200 μM) for 24 h; (**H**) 600 μM FFA and different concentrations (100 and 200 μM) of C3G, (100 and 200 μM) vitisin A, and 1 mM AICAR for 24 h. (**I**) Oil Red O staining of HepG2 cells were cultured with 600 μM FFA and different concentrations (100 and 200 μM) of C3G, (100 and 200 μM) vitisin A, and 1 mM AICAR for 24 h (100×). (**J**) Area calculation of Oil Red O staining. (Values were statistically analyzed using one-way ANOVA between all groups. The results are expressed as means ± SEM of 4 independent experiments. Bars with different letters indicate significant differences, *p* < 0.05. ns, non-significant difference).

Exponentially growing HepG2 cells were treated with varying concentrations of FFA (400, 600, 800, 1000, and 1500 μM) for 24 h, and cytotoxicity was assessed using the CCK-8 assay. FFA concentrations between 400 and 1000 μM did not affect cell viability (Figure 1A). Cells were then treated with different concentrations of C3G (50, 100, 200, 300, and 400 μM), vitisin A (50, 100, 200, 300, and 400 μM), and 5-Aminoimidazole-4-carboxamide1-β-D-ribofuranoside (AICAR) (125, 250, 500, 1000, and 2000 μM). While C3G had no impact on viability across the tested range (Figure 1B), 400 μM vitisin A and 2000 μM AICAR significantly reduced cell viability compared to the blank group (Figure 1C,D).

Treatment with FFA (oleic acid: palmitic acid = 2:1) at concentrations between 400 and 800 μM for 24 h resulted in a significant increase in intracellular TG levels in HepG2 cells, with the most pronounced effects seen at 600 μM and 800 μM FFA (Figure 1E). Vitisin A significantly reduced 600 μM FFA-induced TG accumulation in a dose-dependent manner (50–200 μM) (Figure 1G), whereas C3G only significantly reduced TG levels at 200 μM (Figure 1F).

To directly compare the triglyceride-lowering effects of C3G and vitisin A, HepG2 cells were treated with 600 μM FFA in the presence of 100 μM or 200 μM of either compound, with 1 mM AICAR as a positive control. Both the intracellular TG levels and the quantification of Oil Red O staining demonstrated that at equal concentrations (100 μM and 200 μM), vitisin A was more effective than C3G in reducing TG levels and lipid droplet accumulation (Figure 1H–J). Specifically, compared to the FFA model group, cells treated with 200 μM C3G, 100 μM vitisin A, and 200 μM vitisin A exhibited significant reductions in TG levels by 31.9%, 39.1%, and 44.7%, respectively. These findings indicate that vitisin A has superior triglyceride-lowering activity compared to C3G at equivalent doses in HepG2 cells.

### 2.2. Effect of Vitisin A on Body Weight, Plasma, and Liver Parameters in ApoE^−/−^ Mice

**Figure 2:**
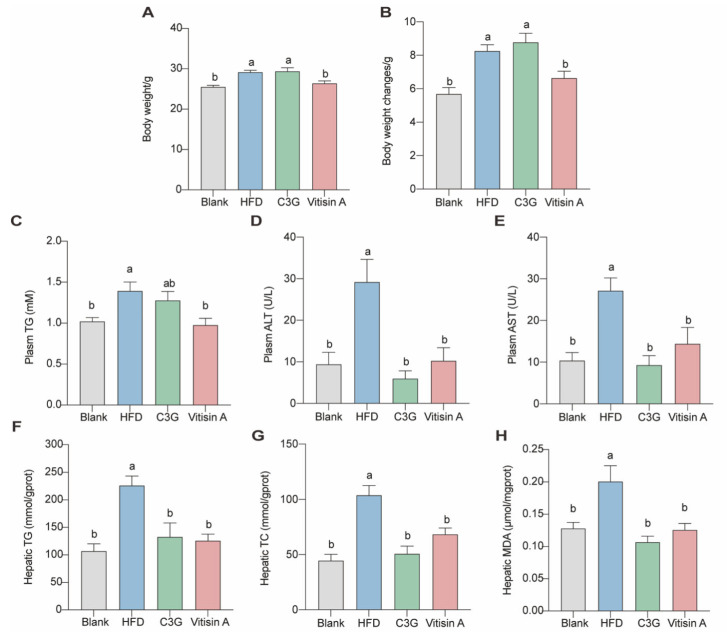
Body weight, Plasma Parameters, and Liver parameters in ApoE^−/−^ mice. Mice subjected to vitisin A presented a reduction in terminal body weight (**A**) and discrepant body weight change (**B**) during 12 weeks of feeding, compared to the model mice. Plasma total TG (**C**), ALT (**D**), and AST (**E**) levels. Liver TG (**F**), TC (**G**), and MDA (**H**) levels. (Values were statistically analyzed using one-way ANOVA between all groups. The results are expressed as means ± SEM of 10 independent experiments. Bars with different letters indicate significant differences, *p* < 0.05).

ApoE^−/−^ mice were treated over 12 weeks, and their body weights were measured periodically. As shown in Figure 2A,B, the high-fat diet (HFD) group saw a significant weight increase, which was reduced by vitisin A. Specifically, vitisin A lowered weight gain by 19.6% (*p* < 0.05), compared to the HFD group, while the C3G group showed no significant difference in weight, compared to the HFD group.

At the end of 12 weeks, plasma TG, aspartate aminotransferase (AST), and alanine aminotransferase (ALT) were measured (Figure 2C–E). Mice in the HFD group had higher levels of total cholesterol (TC) (Figure S1), non-HDL cholesterol, AST, and ALT than those in other groups. Plasma TG levels were reduced by 29.5% in the vitisin A group, compared to the HFD group, but no significant changes were observed in the C3G group. Additionally, ALT and AST levels in the HFD group increased significantly by 211.1% and 161.5%, respectively, compared to the blank group (low-fat diet). Both vitisin A and C3G significantly reduced ALT levels by 64.9% and 79.5%, respectively, while AST levels decreased by 47.0% and 65.8%, respectively. However, neither vitisin A nor C3G affected TC and HDL-C levels. Both compounds also significantly reduced hepatic triglyceride and cholesterol levels (Figure 2F,G), along with reducing liver malondialdehyde (MDA) levels (Figure 2H).

### 2.3. Effect of Vitisin A on NAS Score and Liver Lipid Droplet Accumulation

**Figure 3:**
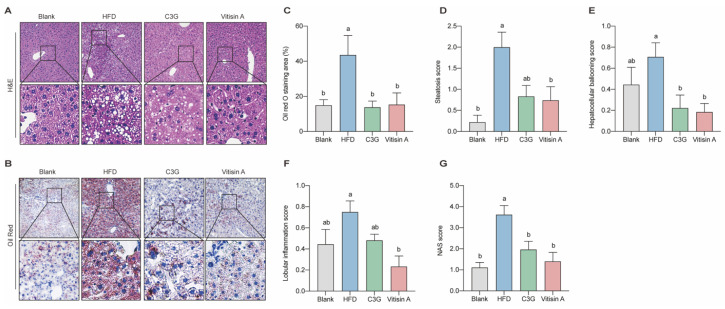
The representative staining histopathology of hepatic results. (**A**) H&E staining (100×). (**B**) Oil Red staining (100×). (**C**) Quantification of the Oil Red O staining area. (**D**) Results of the steatosis score. (**E**) Results of the lobular inflammation score. (**F**) Results of the hepatocellular ballooning score. (**G**) Results of the NAS score. (Values were statistically analyzed using one-way ANOVA between all groups. The results are expressed as means ± SEM of 10 independent experiments. Bars with different letters indicate significant differences, *p* < 0.05).

As expected, mice in the blank group displayed no significant liver steatosis or inflammation, while HFD-fed mice exhibited higher nonalcoholic fatty liver disease activity score (NAS)and significant steatosis. Vitisin A treatment improved these scores, reducing steatosis and hepatocellular ballooning. Similarly, C3G also lowered the hepatocellular ballooning score (Figure 3A,D–G). HFD led to notable lipid accumulation in the liver, which was significantly reduced by both vitisin A and C3G (Figure 3B,C). These findings suggest that vitisin A effectively reduces triglyceride levels in mice. Based on these results, further investigation focused on the underlying mechanisms of vitisin A’s triglyceride-lowering effects is needed.

### 2.4. Effect of Vitisin A on the Fatty Acid Synthesis Pathway in the Liver

**Figure 4:**
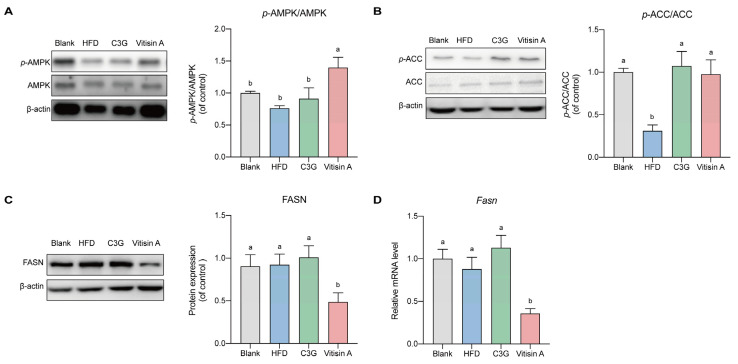
The expression of protein in the liver involved in the FFA synthesis signaling pathway. The representative photographs and grayscale analysis of proteins in the liver involved in the FFA synthesis signaling pathway. (**A**) Protein expression of p-AMPK/AMPK. (**B**) Protein expression of *p*-ACC/ACC. (**C**) Protein expression of FASN. (**D**) mRNA expressions of FASN. (Values were statistically analyzed using one-way ANOVA between all groups. The results are expressed as means ± SEM of 6 independent experiments. Bars with different letters indicate significant differences, *p* < 0.05).

To understand how vitisin A affects fatty acid synthesis, we examined protein and gene expressions related to this pathway. Vitisin A treatment increased the phospho-5′-AMP-activated protein kinase (*p*-AMPK)/5′-AMP-activated protein kinase (AMPK) ratio, while C3G had no significant impact (Figure 4A). phospho-acetyl coenzyme A carboxylase (*p*-ACC)/ acetyl coenzyme A carboxylase (ACC) expression, but both vitisin A and C3G induced moderate upregulation (Figure 4B). Notably, vitisin A significantly reduced fatty acid synthase (FASN)gene and protein expressions by 59.3% and 47.2%, respectively (*p* < 0.05), while C3G had no significant effect (Figure 4C).

### 2.5. Effect of Vitisin A on the β-Oxidation Pathway in the Liver

**Figure 5:**
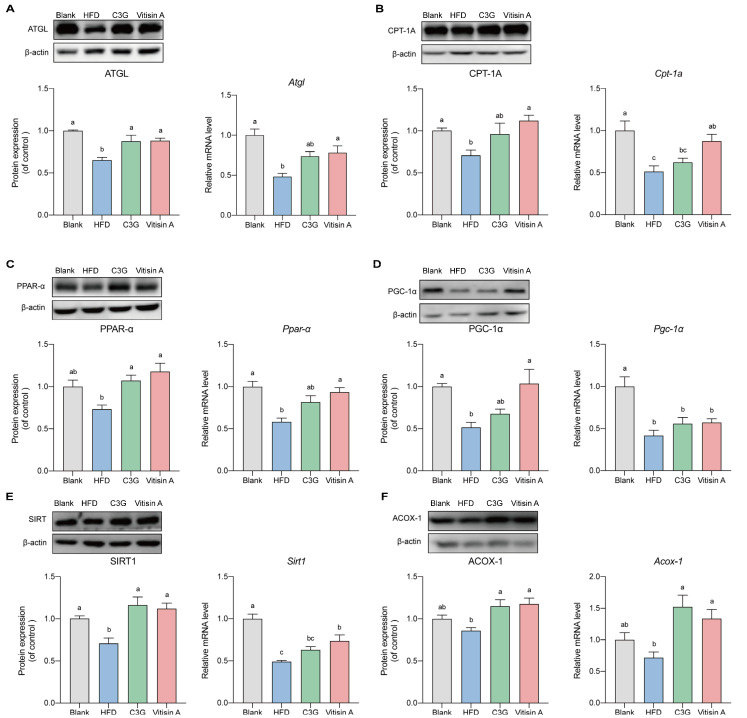
The expression of protein in the liver involved in the β-oxidation signaling pathway. The representative photographs and grayscale analysis of proteins in the liver involved in the β-oxidation signaling pathway. Protein and mRNA expressions of (**A**) ATGL, (**B**) CPT-1A, (**C**) PPAR-α, (**D**) PGC-1α, (**E**) SIRT1, And (**F**) ACOX-1. (Values were statistically analyzed using one-way ANOVA between all groups. The results are expressed as means ± SEM of 6 independent experiments. Bars with different letters indicate significant differences, *p* < 0.05).

The expression of proteins involved in β-oxidation, such as adipose triglyceride lipase (ATGL), carnitine palmitoyl transterase-1A (CPT-1A)peroxisome proliferator-activated receptor-γ coactivator-1 alpha (PGC-1α), andsilent mating-type information regulation 2 homolog 1 (SIRT1), was significantly lower in the HFD group, compared to the blank group, consistent with their mRNA levels (Figure 5A,B,D,E). Vitisin A upregulated the protein and mRNA expressions of ATGL, CPT-1A, PPAR-α, SIRT1, and acyl-CoA oxidase 1 (ACOX-1), compared to the HFD group. The protein expression of PGC-1α was increased by vitisin A. C3G had no significant effect on the protein or mRNA expression of CPT-1A and PGC-1α but increased the protein expression of ATGL, PPAR-α, and SIRT1. The protein and mRNA expressions of ACOX-1 were also increased by treatment with C3G (Figure 5).

## 3. Discussion

Recent epidemiological and genetic studies have established that hypertriglyceridemia is a key factor in the development of cardiovascular diseases, such as atherosclerosis, diabetes, and pancreatitis [19,20,21,22,23]. This underscores the necessity of developing functional foods with effective triglyceride-lowering properties [24,25]. C3G, a common anthocyanin found in dark-colored fruits, vegetables, and grains, has been shown to reduce hepatic and plasma TG levels [26,27,28]. However, in this study, pyranoanthocyanin vitisin A demonstrated superior TG-lowering effects in both in vitro and in vivo models.

In HepG2 cells, vitisin A significantly reduced TG levels and lipid accumulation, compared to C3G at equivalent concentrations. Similarly, in ApoE^−/−^ mice on a high-fat diet, vitisin A reduced body weight gain and lowered plasma and liver TG levels while improving liver histology by decreasing NAS scores and reducing steatosis and hepatocellular ballooning. Both vitisin A and C3G lowered ALT and AST levels, indicating reduced liver damage, though neither compound significantly impacted plasma cholesterol levels.

This study focused on two major pathways through which vitisin A lowers triglyceride levels: inhibition of hepatic DNL and enhancement of fatty acid β-oxidation.

(1) Inhibition of Hepatic DNL via the AMPK/ACC Pathway and Downregulation of FASN

Vitisin A inhibited hepatic DNL by modulating the AMPK/ACC signaling pathway and suppressing the expression of FASN. Under normal conditions, DNL converts excess carbohydrates into fatty acids, which are subsequently esterified into triglycerides for storage [29]. Malonyl-CoA is a key substrate for FASN [30]. ACC catalyzes the conversion of acetyl-CoA into malonyl-CoA, which is then utilized for palmitate synthesis [31,32]. AMPK, a key regulator of energy homeostasis, phosphorylates ACC in serine residues (Ser79/212), inhibiting its activity [33,34]. Dietary anthocyanins from sources such as purple sweet potato, black rice, mulberry, blueberry, Moro orange juice, bilberry, blackcurrant, honeysuckle, and cherry have been shown to modulate AMPK and ACC [16,27,35,36]. Consistent with these findings, our study revealed that C3G upregulated *p*-ACC/ACC but had no significant effect on *p*-AMPK/AMPK. In contrast, vitisin A effectively inhibited DNL in the liver by upregulating both *p*-AMPK/AMPK and *p*-ACC/ACC expressions. In addition to its effects on AMPK and ACC, vitisin A downregulated FASN at both the gene and protein levels. As FASN catalyzed the conversion of malonyl-CoA to palmitate, a precursor for TG synthesis, this reduction in FASN expression mitigated the excessive production of free fatty acids. By contrast, C3G, while increasing *p*-ACC/ACC expression, had no significant impact on AMPK phosphorylation or FASN expression, highlighting the distinct regulatory effects of vitisin A.

(2) Enhancement of Fatty Acid β-Oxidation

Fatty acid β-oxidation is a multi-step process in which fatty acids are broken down to produce energy. Recent studies have identified ATGL as the key enzyme responsible for releasing fatty acids from triglycerides during intracellular lipolysis for energy production. In this study, both C3G and vitisin A (administered at 100 mg/kg bw) increased the expression of ATGL, which facilitated the release of fatty acids from triglycerides during lipolysis. Following lipolysis, long-chain fatty acids are transported into the mitochondria via the carnitine shuttle, where β-oxidation occurs [37,38,39]. Vitisin A significantly upregulated CPT-1A, the rate-limiting enzyme in mitochondrial lipid oxidation, primarily via the activation of the AMPK/SIRT1/PGC-1α and PPAR-α/PGC-1α pathways. While C3G also influenced these pathways, its effects were less pronounced, compared to vitisin A. CPT-1A plays a critical role in the carnitine shuttle, facilitating the transport of long-chain fatty acids into the mitochondria for β-oxidation. AMPK and SIRT1, two pivotal metabolic sensors, regulate PGC-1α activity through phosphorylation and deacetylation, respectively. AMPK-mediated phosphorylation enhances PGC-1α activity, while SIRT1-mediated deacetylation further amplified its transcriptional potential. Consistent with these mechanisms, vitisin A’s ability to enhance AMPK and SIRT1 activities underscores its role in promoting efficient mitochondrial lipid metabolism. PGC-1α, a key transcriptional coactivator, interacts with and activates PPAR-α, which governs the expression of CPT-1A and other genes integral to fatty acid β-oxidation. Previous studies have demonstrated the protective effects of anthocyanin-rich foods, such as bilberry and blackcurrant, against nonalcoholic steatohepatitis and mitochondrial dysfunction via the AMPK/PGC-1α axis. Similarly, dietary anthocyanins derived from table grapes, black rice, blueberry, mulberry, *Hibiscus sabdariffa*, and *Aronia melanocarpa* have been shown to modulate PPAR-α and CPT-1A expressions, corroborating the findings of this study. In addition to mitochondrial oxidation, vitisin A and C3G promoted peroxisomal β-oxidation by upregulating ACOX-1, the rate-limiting enzyme for very-long-chain fatty acid oxidation in peroxisomes. This finding aligns with earlier reports on delphinidin-3-sambubioside, another anthocyanin derivative.

## 4. Materials and Methods

### 4.1. Reagents and Antibodies

C3G (purity > 98%) was isolated from black soybean peels according to the methods described in our previous study [40]. Antibodies for AMPK, *p*-ACC, PGC-1α, SIRT1, and ACOX-1 were purchased from Affinity (Cincinnati, OH, USA). Antibodies for ACC, FASN, ATGL, CPT-1A, proprotein convertase subtilisin/kexin type 9 (PCSK9), and PPAR-α were obtained from Proteintech (Wuhan, China). *p*-AMPK was acquired from Cell Signaling Technology (Boston, MA, USA). Antibodies for 3-hydroxy-3-methylglutaryl coenzyme A reductase (HMGCR) and low-density lipoprotein receptor (LDLR) were obtained from Zenbio (Suzhou, China). The secondary anti-mouse IgG antibody and anti-rabbit IgG antibody were sourced from Proteintech (Wuhan, China). Acetonitrile and formic acid, used for high-performance liquid chromatography (HPLC), were of chromatographic pure grade and were from Merck (Darmstadt, Germany). The AICAR, an AMP-activated protein kinase activator, was obtained from MedChemExpress (Middlesex, NJ, USA). All other chemicals used in this study were of analytical grade unless otherwise specified.

### 4.2. Vitisin A Preparation and Purification

**Figure 6:**
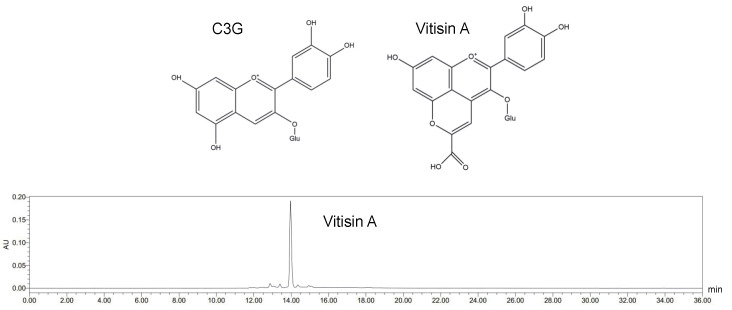
Chemical structure of C3G and HPLC chromatogram of vitisin A.

Vitisin A was obtained as previously described [41]. In brief, vitisin A was synthesized from C3G and pyruvic acid (Macklin, Shanghai, China) in 10 L of phosphate-buffered solution (pH 2.5) at 25 °C in the dark for 45 days, using a molar ratio of C3G to pyruvic acid of 1:100 [42]. The synthetic vitisin A was first processed using a macroporous resin, followed by separation via preparative medium-pressure liquid chromatography. Chromatographic analysis was performed using a C18 column (100 mm × 2.1 mm, 1.8 μm; Agela & Phenomenex, Tianjin, China) at a flow rate of 0.3 mL/min. The mobile phases consisted of solvent A (2% (*v*/*v*) formic acid) and solvent B (acetonitrile). The elution procedure is detailed in Supporting Table S1. The injection volume of the sample was 10 μL. As shown in Figure 6, the isolated compound was identified as vitisin A via HPLC, with a purity of 91.4%, as determined by peak area normalization [12].

### 4.3. In Vitro Study

#### 4.3.1. Cell Culture and Treatments

Human hepatocellular carcinoma cells (HepG2) were purchased from the Cell Library of the Chinese Academy of Sciences. Cells were cultured in DMEM (Sigma-Aldrich, Saint Louis, MO, USA) supplemented with 10% (*v*/*v*) fetal bovine serum (Gibco, Lofer, Austria) and 1% penicillin/streptomycin solution (100 U/mL penicillin, 100 μg/mL streptomycin, Gibco, Lofer, Austria). The cells were maintained at 37 °C in a humidified atmosphere with 5% CO_2_.

#### 4.3.2. CCK-8 Assay

Cytotoxicity was assessed using the Cell Counting Kit-8 (CCK-8) (Beyotime Biotechnology, Shanghai, China) following the manufacturer’s instructions and optimized protocols, reaction time was 60 min.

#### 4.3.3. Triglyceride Determination in Cells

HepG2 cells were seeded into 6-well plates at a density of 4 × 10^5^ cells/well and incubated for 12 h. Cells were then treated with a 600 μM FFA mixture (oleic acid: palmitic acid = 2:1) and various concentrations (0, 50, 100, and 200 μM) of C3G or vitisin A for 24 h. AICAR (1 mM), an AMPK activator, was used as a positive control. Cellular triglyceride levels were measured using a total triglyceride assay kit (Nanjing Jiancheng Bioengineering Institute, Nanjing, China) according to the manufacturer’s protocol.

#### 4.3.4. Oil Red O Staining in HepG2 Cells

HepG2 cells were seeded into 24-well plates at a density of 7 × 10^4^ cells/well and incubated for 12 h. Cells were then treated with 500 μL of 600 μM FFA and various concentrations (0, 100, and 200 μM) of C3G or vitisin A for 24 h. AICAR (1 mM) served as a positive control. Oil Red O staining was performed using a staining kit (Nanjing Jiancheng Bioengineering Institute, Nanjing, China) following the manufacturer’s instructions [43]. Densitometric quantification was carried out using Image J software (1.8.0).

### 4.4. Animals and Treatment

A total of 40 male ApoE^−/−^ mice (4 weeks old) were purchased from GemPharmatech (Nanjing, China) for this experiment. The study was approved by the Animal Care and Protection Committee of Jinan University and adhered to the guidelines for the care and use of laboratory animals. Mice were housed in a controlled environment at 23 °C with a 12 h light/dark cycle. Following a 14-day acclimatization period, the mice were randomly divided into four groups (*n* = 10 per group): blank group, fed a normal chow diet (Catalog # H110010, Beijing Hfk Biosicence, Beijing, China) and given a vehicle via gavage; HFD group, fed a high-fat Western diet (41% energy from fat) with added cholesterol (0.15%, Catalog # H10141, Beijing Hfk Biosicence, Beijing, China) and given a vehicle via gavage; C3G group, fed the high-fat diet and administered 100 mg/kg body weight (bw) of C3G via gavage; and vitisin A group, fed the high-fat diet and administered 100 mg/kg bw of vitisin A via gavage. Based on previous studies, the selected dose of 100 mg/kg bw is safe and suitable for evaluating the bioactivity of anthocyanins in vivo, being slightly above the recommended nutrient intake [12,44]. After 12 weeks of treatment, mice were anesthetized, and blood samples were collected via orbital exsanguination. Organs were harvested, rinsed with saline, and weighed. Portions of the liver were fixed in 4% paraformaldehyde for histological examination, while the remaining tissues were stored at −80 °C for protein, mRNA, and other analyses.

### 4.5. Biochemical Analyses

TG, TC, ALT, AST, HDL-C, and MDA were measured using assay kits from the Nanjing Jiancheng Bioengineering Institute (Nanjing, China), following the manufacturer’s protocols. Non-high-density lipoprotein cholesterol (non-HDL-C) was calculated by subtracting HDL-C from TC.

### 4.6. Histopathological Examination of Hepatic Tissue, NAS Score Evaluation, and Hepatic Lipid Droplet Analysis

Liver samples fixed in 4% paraformaldehyde were dehydrated, embedded in paraffin, and sectioned into 5 μm slices. Histopathological features of the liver, including steatosis, inflammation, and hepatocyte ballooning, were assessed using the NAS system. A double-blinded analysis was conducted to evaluate the extent of NAFLD in the mice. Liver sections stained with Oil Red O were examined under a microscope (Olympus, Tokyo, Japan), and ImageJ software (1.8.0) was used for the densitometric quantification of lipid droplets.

### 4.7. RNA Extraction and Gene Expression Assays

Total RNA was extracted from liver tissue using the HiPure Total RNA Mini Kit (Magen, Guangzhou, China) according to the manufacturer’s instructions. The RNA was then converted to complementary DNA (cDNA). Quantification of hepatic mRNA levels for *Fasn*, *Pgc-1α*, *Sirt1*, *Acox-1*, *Atgl*, *Cpt-1a*, and *Ppar-α* was performed using real-time PCR with TB Green Premix Ex Taq II (TaKaRa, Osaka, Japan) [45]. The PCR amplification cycles were as follows: 95 °C for 30 s, 95 °C for 15 s, and 60 °C for 30 s (45 cycles). The relative mRNA levels were measured after normalizing to β-actin expression. Primer sequences used in this study are provided in Supporting Table S2.

### 4.8. Protein Extraction and Western Blot

Liver tissue was homogenized in lysis buffer (phenylmethanesulfonyl fluoride: RIPA: cocktail of protease inhibitors = 1:100:1). After homogenization, the supernatant was collected by centrifugation at 13,000× *g* for 15 min at 4 °C. Protein concentration was determined using the BCA Protein Assay kit (Beyotime Biotech). Protein samples were then diluted to a concentration of 2.5 g/L using protein loading buffer and PBS. Total liver proteins were separated by sodium dodecyl sulfate-polyacrylamide gel electrophoresis (SDS-PAGE) and transferred onto a PVDF membrane. Western blot analysis was performed using standard protocols established in our previous study. The following primary antibodies were used: p-AMPK (1:500; Cat# 83924-1-RR, Proteintech, Wuhan, China), AMPK (1:1000; Cat# AB32047, Abcam, Shanghai, China), p-ACC (1:1000; Cat# AF3421, Affinity, Suzhou, Chi-na), ACC (1:1000; Cat# 21923-1-AP, Proteintech, Wuhan, China), FASN (1:1000; Cat# 10624-2-AP, Proteintech, Wuhan, China), PGC-1α (1:1000; Cat# AF5395, Affinity, Suzhou, China), SIRT1 (1:1000; Cat# DF6033, Affinity, Suzhou, China), ACOX-1 (1:1000; Cat# DF12046, Affinity, Suzhou, China), ATGL (1:1000; Cat# 55190-1-AP, Proteintech, Wuhan, China), CPT-1A (1:1000; Cat# 15184-1-AP, Proteintech, Wuhan, China), PPAR-α (1:1000; Cat# 15540-1-AP, Proteintech, Wuhan, China), LDLR (1:1000; Cat# R380860, Zenbio, Su-zhou, China), HMGCR (1:1000; Cat# R24588 Zenbio, Suzhou, China), PCSK9 (1:500; Cat# 55206-1-AP, Proteintech, Wuhan, China) [46]. 

### 4.9. Statistical Analysis

Data were expressed as mean ± standard error of mean (SEM). Statistical analysis was performed using one-way analysis of variance (ANOVA) followed by Fisher’s least significant difference test. The value of *p* < 0.05 was considered statistically significant.

## 5. Conclusions

In conclusion, vitisin A exerts superior triglyceride-lowering effects, compared to C3G, through dual mechanisms involving the inhibition of hepatic DNL and the enhancement of fatty acid β-oxidation. Vitisin A effectively downregulates DNL by activating the AMPK/ACC signaling pathway and suppressing FASN expression, a regulation that was not observed with C3G. In parallel, vitisin A robustly enhances fatty acid β-oxidation by upregulating key enzymes across mitochondrial and peroxisomal pathways. It significantly promotes CPT-1A expression through the AMPK/SIRT1/PGC-1α and PPAR-α/PGC-1α pathways, facilitating efficient mitochondrial lipid oxidation. Additionally, both vitisin A and C3G upregulate ACOX-1, driving the peroxisomal β-oxidation of very-long-chain fatty acids. These findings highlight vitisin A’s ability to comprehensively target multiple lipid metabolism pathways, distinguishing it as a promising candidate for managing hypertriglyceridemia and related metabolic disorders. Currently, apoE^-/-^ mice are used to test the efficacy of pharmacological interventions in human study^49^. However, whether this mice models accurately mimic human disease is open to discussion. Substantial differences exist between humans and mice in lesions dis- tribution and progression [47].

## Data Availability

Data will be available upon request from the corresponding author.

## Supplementary Materials

The following supporting information can be downloaded at: https://www.mdpi.com/article/10.3390/ijms26041521/s1.
