# Supplementary material for: Vitisin A Outperforms Cyanidin-3-O-Glucoside in Triglyceride Reduction by Modulating Hepatic Lipogenesis and Fatty Acid β-Oxidation
Source: Int J Mol Sci. 2025 Feb 11;26(4):1521. doi: 10.3390/ijms26041521 (PMC11855501; doi:10.3390/ijms26041521)
Supplement: Supplementary file 1 [file ijms-26-01521-s001.zip › ijms-3457726-supplementary.pdf]

Supporting Table S1 The elution procedure of medium-pressure liquid chromatography

Time/min	B%
10	6-6
10	6-15
40	15-32
40	32-33
40	33-34
40	34-35
20	35-37
20	37-44
20	44-81
10	81-100
50	100-100
10	100-50
10	50-6
5	6-6

Supporting Table S2 Primer sequences used for real-time PCR analysis

Gene name	Accession No.	Primer sequence (5'→3')	
Cpt-1a	NM_0134952	Forward	AAGTGTGGAACTCTCTGGAACTG
		Reverse	GGGTTATCTTGGTTGGCTTTATG
Ppar-α	NM_001113418	Forward	GGGCAAGAGAATCCACGAAG
		Reverse	GTTGTTGCTGGTCTTTCCCG
Atgl	NM_001163689	Forward	TTCACCATCCGCTTGTTG
		Reverse	AGTTCCACCTGCTCAGAC
Sirt1	NM_001159589	Forward	GCAGGTTGCAGGAATCCAA
		Reverse	GGCAAGATGCTGTTGCAAA
Pgc-1α	NM_008904	Forward	AAGTGTGGAACTCTCTGGAACTG
		Reverse	GGGTTATCTTGGTTGGCTTTATG
Fasn	NM_0079883	Forward	GTGTGGACATGGTCACAGATG
		Reverse	GACCGCTTGGGTAATCCATA
Acox-1	NM_001271898	Forward	CCTGATTCAGCAAGGTAGGG
		Reverse	TCGCAGACCCTGAAGAAATC
β-actin	NM_007393	Forward	CGTGAAAAGATGACCCAGATCA
		Reverse	CAGCCTGGATGGCTACGTACA

Supporting Figure S1 Effect of C3G and Vitisin A on plasm total TC level and the expression of protein involved in cholesterol metabolism in ApoE$^{-/-}$ mice.

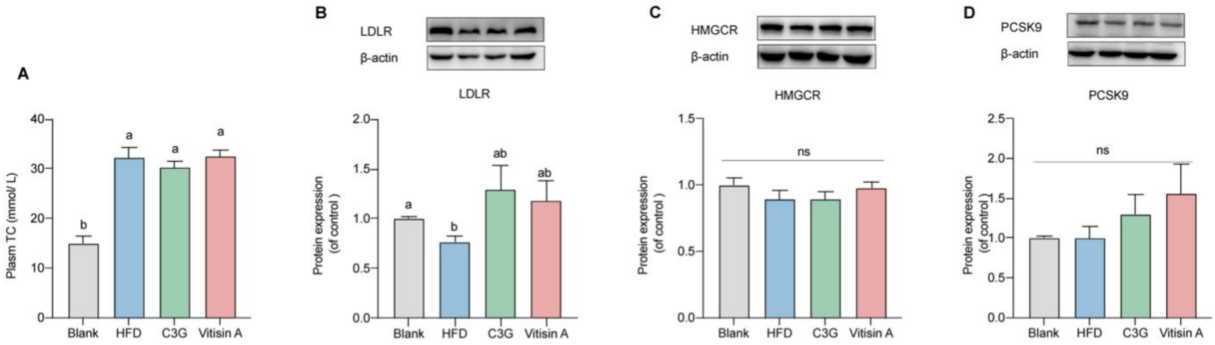

Supporting Figure S1 Effect of C3G and Vitisin A on TC level in ApoE$^{-/-}$ mice.

(A) Mice subjected to C3G and Vitisin A during 12 week feeding. Plasma total TC level. The representative photographs and grayscale analysis of proteins in the liver involved in cholesterol metabolism. Protein expression of (B) LDLR. (C) HMGCR. (D) PCSK9. (Values were statistically analyzed using One-way ANOVA between all groups. The results are expressed as means ± SEM of 6 independent experiments. Bar with different letters indicate significant differences, $p < 0.05$. ns, non-significant difference.).
